# Associations between Caries among Children and Household Sugar Procurement, Exposure to Fluoridated Water and Socioeconomic Indicators in the Brazilian Capital Cities

**DOI:** 10.1155/2013/492790

**Published:** 2013-11-07

**Authors:** Michele Martins Gonçalves, Cláudio Rodrigues Leles, Maria do Carmo Matias Freire

**Affiliations:** Federal University of Goias, School of Dentistry, 74605-220 Goiania, GO, Brazil

## Abstract

The objective of this ecological study was to investigate the association between caries experience in 5- and 12-year-old Brazilian children in 2010 and household sugar procurement in 2003 and the effects of exposure to water fluoridation and socioeconomic indicators. Sample units were all 27 Brazilian capital cities. Data were obtained from the National Surveys of Oral Health; the National Household Food Budget Survey; and the United Nations Program for Development. Data analysis included correlation coefficients, exploratory factor analysis, and linear regression. There were significant negative associations between caries experience and procurement of confectionery, fluoridated water, HDI, and per capita income. Procurement of confectionery and soft drinks was positively associated with HDI and per capita income. Exploratory factor analysis grouped the independent variables by reducing highly correlated variables into two uncorrelated component variables that explained 86.1% of total variance. The first component included income, HDI, water fluoridation, and procurement of confectionery, while the second included free sugar and procurement of soft drinks. Multiple regression analysis showed that caries is associated with the first component. Caries experience was associated with better socioeconomic indicators of a city and exposure to fluoridated water, which may affect the impact of sugars on the disease.

## 1. Introduction

There is a correlation between sugar consumption and caries experience in children using population data from different countries [1–3]. Exposure to fluorides in public water supplies and fluoridated toothpastes are considered to be the main reasons for the reduction in the prevalence and inequalities in caries distribution [4, 5] and may influence the impact of sugar consumption on development of caries in children [6]. 

Ecological studies are useful to identify relationships between population-level exposures to risk factors and population-level outcomes using aggregated data [7]. In Brazil, few studies have used such an approach to explore the effects of population-based variables on caries distribution [8–10]. The latest Brazilian oral health national surveys carried out in 2003 and 2010 showed that there were marked inequalities in the distribution of caries, with higher levels of the disease and treatment needs in lower socioeconomic groups [11, 12]. Further analysis of factors associated to caries prevalence from the 2003 Brazilian national survey demonstrated the role of socioeconomic factors and exposure to fluoridated water on caries distribution [13, 14].

Although there is sound evidence that dental caries is significantly influenced by sugars consumption at the individual level using dietary inventories [6, 15], the influence on caries prevalence of sugar procurement (acquisition from an external source) at the population level and how fluoridation of drinking water and socioeconomic factors affect any such association are not clear. Also, previous ecological studies were carried out between countries and the possible relationships within a country have not been investigated. The results are useful to increase the knowledge on the inequalities in dental caries distribution between cities in a country. In our previous study with this objective and using data on caries and sugar availability collected in the same year (2003), there was no association between dental caries in children and sugar availability in the Brazilian capital cities when socioeconomic indicators and presence of fluoridated water were considered in the analysis [16]. It is now hypothesized that this result would be different using data on caries experience collected seven years after data collection for sugar procurement, which would be more appropriate to indicate a possible effect of diet on the disease. Therefore, the objective of this ecological study was to investigate the effects of exposure to water fluoridation and socioeconomic indicators on the association between caries experience in 5- and 12-year-old Brazilian children in 2010 and household sugar procurement in 2003.

## 2. Methods

### 2.1. Databases and Aggregated Measurements

This study included data on caries prevalence at ages 5 and 12, household procurement of free sugar, confectionery and sugary soft drinks, exposure to fluoridated water, and socioeconomic and development indicators (Human Development Index—HDI, per capita income and Gini Index). 

Dental caries prevalence data was obtained from the 2010 Brazilian National Survey of Oral Health [12]. Sample size was calculated to be representative of the country, its five national geographic regions, and the capital cities of all Brazilian states. In the present study we selected only data from the 27 capital cities, whose primary sample units were the census sectors used by the Brazilian Institute of Geography and Statistics (IBGE), which fulfilled criteria for the deployment of data collection covering a specific range of households in an urban area. Sampling was based on a probability scheme implemented as a multistage sampling containing all state capitals and the Federal capital city, as well as other randomly selected inner cities stratified by population size in each state. Data were collected through oral clinical examinations and interviews on a sample of 37,519 individuals. Aggregated measurements of dental caries experience at ages 5 and 12 were the main outcome for the purpose of this study. Caries indexes were the DMFT/dmft and percentage of caries-free children (DMFT/dmft = 0), according to criteria proposed by the World Health Organization [18]. 

Data about procurement of free sugars and sugary products (confectionery and soft drinks) were retrieved from the 2003 Household Food Budget Survey (HFBS) of the IBGE [17] on a sample of 48,470 households. These are governmental surveys periodically performed to measure the patterns of household consumption expenditures. The HFBS was planned to be representative of households in Brazil as a whole. Sampling was organized in a two-stage cluster design, stratified by geographic location and census sectors by IBGE in the year 2000, and households were selected by simple random sampling within the sectors. In each household, during seven consecutive days, all food and beverages purchased for family consumption were recorded. Aggregated data on sugar and sugar-containing foods for household consumption were recorded and expressed as kilograms per capita per year. Specific listed items were free sugar (sucrose) added to foods by the consumer, all types of soft drinks, and all types of confectionery ready for consumption, for example, candies, gums, chocolates, popsicle, and ice cream. 

For the purpose of the present study, items were pooled into three category groups: free sugar, sugary soft drinks and confectionery, and the quantity of purchased foods transformed into per capita annual procurement by each category. For estimation of annual procurement, the seven-day procurement of each category was divided by the total number of household members and multiplied by an annual factor of 52 to estimate the purchases for 364 days (one year).

Information on fluoridation of public water supplies in the capital cities and time in years since fluoridation were obtained from the Brazilian National Survey of Oral Health in 2003 [11].

Socioeconomic indicators of the Brazilian capital cities were retrieved from the Atlas of Human Development for the year 2000, by the United Nations Program for Development [19]. Specific indicators included the Human Development Index (HDI), the Gini Index, and income per capita. HDI measures the degree of economic development and quality of life of the population, considering education (literacy and enrollment rates), longevity (life expectancy of the population), and income (GDP per capita). HDI ranges from 0 (no human development) to 1 (total human development). The Gini Index is a measure of inequality in the income distribution, indicating the extent to which the distribution of income among individuals or households deviates from a perfectly equal distribution. Income per capita represents the sum of the salaries of the entire population divided by the number of inhabitants within the city, expressed by the Brazilian currency.

### 2.2. Data Analysis

All databases with aggregated data were checked and cleaned separately to include only the cities and variables selected for the present study. Descriptive analysis of caries experience for the 5- and 12-year-old groups was performed using mean values for dmft/DMFT scores and percentages for caries prevalence. Means, minimum, and maximum values were also provided for the five Brazilian geographical regions. In order to facilitate presentation of descriptive data, we calculated the mean scores for each region by summing up the values of all capital cities in that region and dividing the result by the number of cities. However, for further data analysis the unit of analysis was the city. All other variables were expressed as absolute aggregated values.

The Kolmogorov-Smirnov test was used to test normality of data distribution and, except for sugar procurement, all variables had a normal distribution. Pearson and Spearman's correlation coefficients were used to test bivariate association between caries experience (dependent variable) and the independent variables (sugar procurement, water fluoridation, and socioeconomic indicators). Correlations between the independent variables were also tested. All correlation analysis were performed at the capital city level (*n* = 27).

Exploratory factor analysis was used for reduction of variables, considering the small number of observations (*n* = 27) in relation to the high number of independent variables, and also the fact that most of the independent variables were expected to be highly intercorrelated. Analysis was based on eigenvalues greater than 1, with varimax rotation. Hence, multiple linear regression analysis would not be recommended due to the problem of multicollinearity. In order to manage this problem, independent variables were combined into joint variables running an exploratory factor analysis to group variables by reducing the highly correlated variables into uncorrelated “component variables.” This method combined the variables that load on the same factor and, subsequently, using a principal components regression by using the factor scores saved as new variables as the values of the component variables.

The components derived from factor analysis were used as independent variables to test the association with caries experience variables using multiple linear regression. Significance level was set at *P* < 0.05 for all analyses. SPSS 17.0 software (SPSS, Inc., Chicago, EUA) was used for database construction and statistical analysis.

## 3. Results

**Figure 1:**
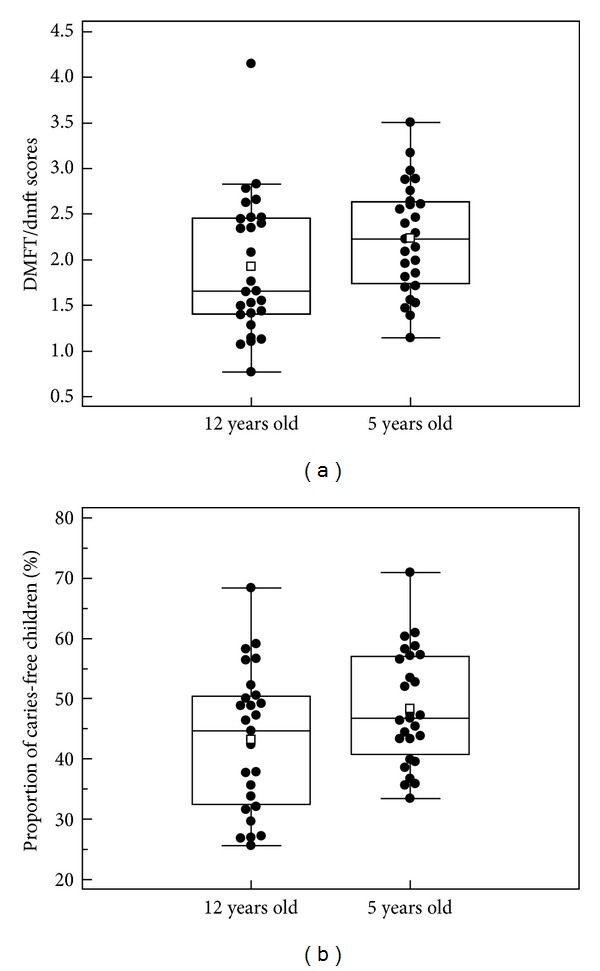
Data distribution of caries experience (a) and proportion of caries-free children (b) in the Brazilian capital cities. The blank squares are mean values.

Figure 1 shows the distribution of caries experience of the 27 Brazilian capital cities in 2010. Mean dmft/DMFT (SD) scores for the total sample were 2.23 (0.60) and 1.92 (0.76) for 5- and 12-year-old children, respectively. The mean percentages of caries-free children were 48.4% (SD = 9.5) at age of 5 and 43.2% (SD = 11.6) at age of 12. 

Table 1 summarizes data on caries experience (DMFT/dmft scores and percentage of caries-free children) for both age groups according to the five geographic regions of Brazil: North (*n* = 7), Northeast (*n* = 9), Central-West (*n* = 4), Southeast (*n* = 4), and South (*n* = 3). The same summary statistics were detailed in Table 2 for measures of sugar procurement in 2003, socioeconomic variables, and water fluoridation in the capital cities, according to the five regions.

Correlation analysis in Table 3 showed significant negative associations between caries experience (percent of caries-free children and DMFT/dmft index) and procurement of confectionery, exposure to fluoridated water, HDI, and per capita income. Procurement of confectionery and sugary soft drinks sugar were positively associated with HDI and per capita income, while procurement of free sugar was not correlated with any of the socioeconomic variables studied (*P* < 0.001). The Gini Index was excluded from all further analysis due to the almost absolute correlation with each of the other variables.

Factor analysis gave two components that explained 86.1% of the total variance.  Table 4 presents the rotated component matrix which contains the rotated loadings for each variable, sorted by order, which classify the variables into the component that has the greatest loading value. Component 1 included income, HDI, water fluoridation, and confectionery procurement, while Component 2 included free sugar and sugary soft drinks procurement. As an assumption of factor analysis, rotation of these components generate loading scores that are uncorrelated (*r* = 0.00; *P* = 1.000). This means that components represented by free sugar and soft drinks procurement have no association with Component 1.

Multiple regression analysis (Table 5) showed that both DMFT/dmft scores and caries-free prevalence were associated with Component 1, which encompasses variables related to socioeconomic and development conditions. Regression coefficients for Component 1 were significant for all measures of caries experience and greater for the 12-year-old group (*P* < 0.001 and *R* ^2^ values greater than 40%) than for the 5-year-old groups.

## 4. Discussion

This study showed that children's caries experience in 2010 was associated with better socioeconomic indicators of cities and exposure to fluoridated water. In addition, there was no association between caries and procurement of free sugar, confectioneries, and sugary soft drinks in 2003, after controlling for other factors. Similar results were found in our previous study using data on sugar procurement and dental caries experience collected in the same year (2003) [16]. These findings were unexpected since there is sound evidence on the direct relation between intake of dietary sugars and dental caries across the life span [6, 15]. Therefore, results of the present study have to be interpreted considering a number of methodological issues. 

Availability of confectionery and sugary soft drinks was measured by their purchase in households, which is an indicator of food procurement by the families but is not necessarily a measure of their consumption pattern. The procurement of these manufactured items, but not the procurement of free sugar, was also correlated with socioeconomic variables, indicating that families with better living conditions tended to purchase more consumer goods, including sugary foods [20]. 

Evidence from other ecological studies on the sugar-caries relationship has shown conflicting results. Positive correlation between the availability of sugar and dental caries in 12-year-old children was found in studies involving international comparisons [1–3, 21]. However, this association was not found when only developed countries were analyzed [2]. In addition, Downer et al. [22] found that lower caries prevalence in 29 European countries was associated with higher levels of sugar consumption and also better socioeconomic conditions. These findings were in part attributed to the effect of fluoridated toothpaste on the reduction of the incidence of caries [22]. Recent research has also shown that the relationship between per capita consumption of sugar and dental caries is modified by the absolute level of income of the country [3]. 

Another ecological study that included 27 European countries and Israel, Canada, and the United States showed that low rates of regular toothbrushing and high consumption of sweets were associated with higher DMFT scores, while high rates of soft drink consumption were associated with lower DMFT scores [23]. The probable explanation for these findings was the economic condition of the studied countries; for those with high caries levels the consumption of soft drink was still increasing. This may be also true for Brazil, since the negative correlation between procurement of confectioneries and caries experience found initially in the bivariate analysis did not remain significant after controlling for other factors. There is also evidence from the national Household Food Budget Surveys that the overall household income has a substantial effect on the procurement of most foods and sugars [24]. Our results suggest that data on household food availability, such as those from the Brazilian surveys, may be not appropriate to evaluate the population consumption of sugary products because, as suggested by Sreebny [1], they are more correlated to expenditures on food. We, therefore, recommend the inclusion of data on individual sugars consumption and other oral health related behaviours in the Brazilian oral health national surveys, which would allow for more appropriate analysis considering both individual and contextual levels. 

Limitations and risk of bias also include the time lapse between the oral health survey and the dietary survey, carried out at different time points, which may have introduced some degree of variation in the measurement. Other factors include the lack of data on food consumed outside the home, the short period (one week) for the recording of food purchases in the household enquiry, and the unknown proportion of purchased foods that was not consumed. An unavoidable shortcoming is that, whereas the data on caries experience refer only to the 5- and 12-year-old children, the other data relates to whole populations in the participating cities. Also, the sample size was small to allow for more appropriate statistical analysis, although representative of all 27 Brazilian capital cities.

The high availability of sugars and sugary foods and drinks in Brazil [24] draws attention to the role of sugars, especially sucrose, as an important risk factor for many chronic nontransmissible diseases, besides dental caries. The National School-Based Health Survey carried out in Brazil in 2009 [25] showed that 58.3% of the adolescents reported eating confectionery and 37% reported drinking soft drinks on five or more days a week. That consumption pattern was positively associated with reported prevalence of dental pain [26]. In addition, soft drinks may also contribute to dental erosion [27]. Healthy eating strategies have been implemented as part of public health policies in Brazil, seeking to reduce health problems associated with the high consumption of sugars, fats, and salt [28].

Information about sugars consumption in the population is not systematically collected in Brazil. The high cost and excessive operational work make it difficult to carry out dietary surveys, which are considered the most appropriate method to measure food intake [29]. Thus, information on dietary intake has been indirectly estimated from the household food availability, measured by the procurement of food and drinks [18, 24]. 

The association between socioeconomic status and dental caries was previously shown in studies using different populations, study designs, and socioeconomic variables [30, 31]. In our study, the HDI and per capita income were inversely associated with caries experience, as reported in previous studies in Brazil [8, 32] and elsewhere [30]. Although some studies showed a positive correlation between the Gini coefficient and caries experience [10, 33, 34], we did not find significant associations between these variables. A similar finding was reported for children aged 5-6 in Sao Paulo, Brazil [32]. A possible explanation is the low variability in Gini coefficients among the Brazilian state capitals, since they had similar patterns of income distribution. According to Wilkinson [35], the inequality in income distribution has a greater effect for more heterogeneous populations and, when comparing populations with more homogeneous levels of socioeconomic inequality, the effect of this variable is smaller or even null when controlled by total income. Also, a study carried out in Brazil showed that income inequality effect on dental caries among adolescents was explained mainly by municipal public policies, which had an independent effect that was greater among the better-off [36].

The significant negative correlation found in our study between the time of exposure to community fluoridated water and caries experience is consistent with results from other population-based studies in Brazil [8, 32, 37, 38]. Inequalities in water fluoridation among Brazilian municipalities were also found in the national oral health survey in 2003, suggesting an association between fluoridation and socioeconomic variables [9]. The most favorable conditions were found in cities with larger populations, situated in more economically developed regions and with higher HDI.

The results of the present study confirm that oral health is associated with social and economic indicators at the population level. This is important to guide public health policies to mitigate health inequities among populations of different socioeconomic conditions. Further research is needed to clarify the associations between dental caries and the availability of sugars and sugary foods and drinks, as well as the probable protective effect of the availability of fluoride toothpastes on caries experience of Brazilian populations. Periodical assessment to monitor changes in food procurement and consumption within population groups is essential to evaluate dietary habits and identify potential determinants of health with implications for oral and dental conditions.

## Figures and Tables

**Table 1:** Mean values of caries experience in the Brazilian capitals in 2010, according to the five geographic regions. Minimum and maximum values are in parenthesis.

Brazilian geographic regions	dmft in 5-year-old children	DMFT in 12-year-old children	% caries-free 5-year-olds	% caries-free 12-year-olds
North (*n* = 7)	2.65 (1.53–3.51)	2.74 (2.34–4.15)	41.1 (33.4–53.5)	29.7 (25.6–35.6)
Northeast (*n* = 9)	2.16 (1.39–2.76)	1.87 (1.07–2.78)	48.5 (39.5–58.7)	44.2 (29.6–59.1)
Central-West (*n* = 4)	2.39 (1.85–3.17)	1.73 (1.14–2.40)	46.3 (35.9–52.8)	46.7 (37.7–56.6)
Southeast (*n* = 4)	1.75 (1.14–2.40)	1.29 (1.10–1.41)	58.0 (45.4–71.0)	52.1 (49.2–56.4)
South (*n* = 3)	1.91 (1.56–2.46)	1.27 (0.77–1.53)	55.0 (43.8–60.9)	54.0 (44.7–68.4)

Total for Brazil (*n* = 27)	2.23 (1.14–3.51)	1.92 (0.77–4.15)	48.4 (33.4–71.0)	43.2 (25.6–68.4)

Data from the 2010 Brazilian National Survey of Oral Health [11].

**Table 2:** Mean values of the sugar procurement, socioeconomic variables, and water fluoridation in Brazilian capitals, according to the five geographic regions of Brazil. Minimum and maximum values are in parenthesis.

	Procurement^1 ^of free sugar	Procurement^1 ^of sugary soft drinks	Procurement^1^ of confectionery	HDI^3^	Gini Index^3^	Per capita income^3^	Water fluoridation (years)^2^
North (*n* = 7)	13.7 (11.9–16.0)	19.3 (9.4–31.1)	0.34 (0.19–0.72)	0.78 (0.75–0.81)	0.63 (0.58–0.65)	293.9 (253.7–358.1)	1 (0–7)
Northeast (*n* = 9)	14.6 (7.3–21.0)	16.3 (7.7–23.1)	0.58 (0.32–1.01)	0.78 (0.74–0.81)	0.65 (0.63–0.68)	317.1 (250.7–392.5)	4.3 (0–14)
Central-West (*n* = 4)	23.1 (13.5–46.2)	39.1 (22.6–70.5)	2.30 (0.82–4.99)	0.83 (0.81–0.84)	0.63 (0.61–0.65)	487.6 (394.7–605.4)	14.3 (0–26)
Southeast (*n* = 4)	15.2 (11.2–19.5)	33.4 (23.1–42.5)	1.79 (1.38–2.28)	0.84 (0.84–0.86)	0.62 (0.61–0.62)	608.0 (557.4–667.7)	22.0 (16–28)
South (*n* = 3)	11.6 (8.1–15.6)	36.7 (27.4–46.1)	2.34 (2.05–2.51)	0.87 (0.86–0.88)	0.59 (0.57–0.61)	677.0 (619.8–709.9)	30.3 (21–44)

Brazil (*n* = 27)	15.4 (7.3–46.2)	25.2 (7.7–70.5)	1.15 (0.19–4.99)	0.81 (0.74–0.88)	0.63 (0.57–0.68)	419.4 (250.7–709.9)	10.4 (0–44)

Data from the ^1^2003 Household Food Budget Survey  [16], ^2^2010 Brazilian National Survey on Oral Health [11], and ^3^2000 United Nations Program for Development [17].

**Table 3:** Correlation coefficients of the association between caries experience in 5- and 12-year-old children in 2010 and the independent variables.

Caries	Age groups	Sugar procurement			Water fluoridation	Socioeconomic indicators		
		Free sugar	Sugary soft drinks	Confectionery		Gini Index	HDI	Per capita income
DMFT	5-years-old	−0.16	−0.32	−0.39*	−0.41*	−0.08	−0.54**	−0.50**
	12-years-old	−0.23	−0.30	−0.51**	−0.64**	0.10	−0.67**	−0.62**
% caries-free	5-years-old	−0.08	0.37	0.42*	0.46*	−0.04	0.60**	0.57**
	12-years-old	0.19	0.38	0.57**	0.59**	−0.10	0.67**	0.65**

*Significant at the *P* < 0.05 level.

**Significant at the *P* < 0.01 level.

**Table 4:** Rotated loadings matrix for the extracted component variables.

	Component*	
	1	2
Per capita income	**0.98**	0.09
HDI	**0.95**	0.10
Water fluoridation	**0.83**	0.12
Confectionery procurement	**0.82**	0.26
Free sugar procurement	0.00	**0.97**
Soft drinks procurement	**0.61**	**0.72**

*Values in bold font represent loading values greater than 0.50. The greatest loadings for each variable indicate in which component the variable is located in the final factor solution.

**Table 5:** Multiple linear regression of the association between caries experience in 5- and 12-year-old children in 2010 and the component variables.

Age groups	Caries experience	Components*	Unstandardized coefficients (95% CI)	*P* value	*R*-square
5 years old	DMFT/dmft score	Component 1	−0.30 (−0.52; −0.08)	0.009*	0.25
		Component 2	−0.04 (−0.26; 0.17)	0.683	
	% caries-free	Component 1	5.43 (2.18; 8.69)	0.002*	0.33
		Component 2	0.07 (−3.19; 3.33)	0.967	
12 years old	DMFT/dmft score	Component 1	−0.49 (−0.73; −0.25)	<0.001*	0.42
		Component 2	−0.04 (−0.28; 0.21)	0.766	
	% caries-free	Component 1	7.61 (3.94; 11.29)	<0.001*	0.44
		Component 2	1.18 (−2.50; 4.85)	0.515	

*Component 1 included income, HDI, water fluoridation, and confectionery procurement; Component 2 included free sugar and sugary soft drinks procurement.
